# Exchanges

**Published:** 1856-04

**Authors:** 


### EXCHANGES.

Many of these come to us with the friendly hint upon the cover to exchange. We are surprised at this, as we have regularly mailed to each and every Medical Journal now published in the country. We have been made to know, with no small regret, that our mail service is most miserably managed, but recent developments have produced in our minds, feelings other than mere regret. The *growlings* of our subscribers and the complaints of our exchanges, the absence of many choice works, regularly published, do not show a commensurate progress in this department of the government, with the increased facilities now at command, and we lose all patience when the evidences of delinquency are so multiplied.

